# Comparative Study on the Outcome of Stroke Patients Transferred by Doctor Helicopters and Ground Ambulances in South Korea: A Retrospective Controlled Study

**DOI:** 10.1155/2020/8493289

**Published:** 2020-11-01

**Authors:** Jeong Il Lee, Kang Hyun Lee, Oh Hyun Kim

**Affiliations:** ^1^National Civil Defense and Disaster Management Training Institute, Gongju City, Chungnam Province, Republic of Korea; ^2^Department of Emergency Medicine, Yonsei University Wonju College of Medicine, Wonju City, Gangwon Province, Republic of Korea

## Abstract

The purpose of this study was to analyze the effectiveness of helicopter emergency medical services (HEMS) for its economic operations in South Korea. This study targeted stroke patients who were transported via HEMS or ground emergency medical services (GEMS) from the scene of an accident to a regional emergency medical center. From this patient population, stroke patients who traveled at least 50 km from the scene of the cerebral infarction to the hospital with analyzable outcome data were extracted and included in this study. This study included 26 HEMS and 102 GEMS stroke patients from a pool of 183 potential patients. The survival-to-discharge rate of patients transported via HEMS (96.2%; 25/26) was significantly higher than that of patients transported via GEMS (83.2%; 104/128) (*P*=0.001). The HEMS transfer was quicker with respect to the decision-making process because the emergency physician actively evaluates and communicates on-site and during in-transit travel to request an appointment immediately upon arrival at the emergency room. These results indicate that using HEMS increased discharge and survival rates and reduced in-hospital mortality of HEMS of stroke patients with a reduced admission time. This result association leads to reasonable cost-effectiveness and efficient estimates overall. In conclusion, HEMS indicate reduced time taken for stroke patients to be hospitalized and treated and decreased mortality after 24 hours. According to this result, HEMS transport can be more effective than GEMS in long-distance delivery of stroke patients.

## 1. Introduction

Stroke is the most frequent cause of permanent disability in adults and one of the most frequent causes of death [1–3]. In addition to substantial individual suffering, stroke results in enormous costs to society [4–6]. Stroke is a medical emergency with a short time window for thrombolytic therapy [7]. Since the introduction of thrombolytic therapy for ischemic stroke, public health authorities and clinical facilities have sought effective ways to reach stroke victims rapidly for evaluation and treatment [8]. Approximately 85,000 people experience a new or recurrent stroke each year, with the cost of stroke care accounting for an estimated $2 trillion in the healthcare system of South Korea [9]. It is difficult to provide effective and high-quality treatment to patients without knowing their diagnosis. Likewise, for healthcare systems to be effective, improvements must be made through ongoing research into health and how these issues are changing [10]. A number of strategies have been shown to improve treatment rates, including rapid recognition by the public and early access to emergency care. However, South Korea has been unable to present solutions or clear guidelines for several problems related to stroke patient transfer.

Since the start of helicopter transportation for domestic emergency medical care in 2011, we are doing doctor helicopter service for five hospitals (Gangwon, Jeonnam, Jeonbuk, Kyunguk, and Chungnam). It has been reported that stroke patients transported via helicopter emergency medical services (HEMS) in Korea have been treated quickly and accurately in the hospital. The operation of doctor helicopters is costly, and their effectiveness for stroke patients in domestic doctor helicopter operations has not yet been studied. In this study, we tried to examine the effectiveness of HEMS and the direction of improvement for the transport of stroke patients in the doctor helicopter system.

## 2. Methods

### 2.1. Study Design

This retrospective cohort study was conducted to investigate the current situation and the effect of the HEMS system for improving emergency medical services since its introduction. The study was conducted from July 2013 to February 2015, targeting stroke patients via HEMS or GEMS. The patient data evaluated included prehospital care, hospital care, and the final outcome. We compared these data in two groups of stroke patients: an HEMS group and a GEMS group.

For comparisons, the stroke patients who were classified according to stroke severity and only those who were transported at least 50 km to an area emergency medical center via HEMS or GEMS were included in the final analysis. Considering the characteristics of the topography, the transfer time over distances of 50 km or more and over mountainous areas is at least 1 hour.

### 2.2. Region

**Figure 1:**
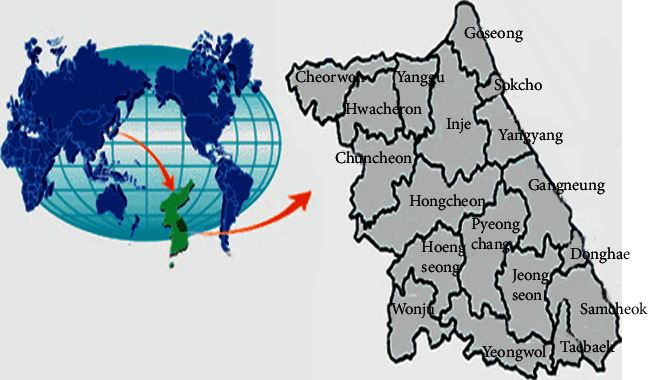
Map of Gangwon Province (accessed at http://www.provin.gangwon.kr/gw/portal).

Gangwon province is located in the mid-eastern part of the Korean peninsula and is divided into two areas, Yeongdong and Yeongseo, by the Taebaek mountains running along the eastern part of the peninsula. It extends between 37°02′ and 38°37′ north latitude and 127°05′ and 129°22′ east longitude, and the 38th parallel crosses the middle of the province. The province is also crossed by the 145 km long Military Demarcation Line (MDL), which starts at 38°45′ north latitude, in Hyeonnae-myeon in Goseong-gun to the southwest, to a site at 38°20′ north latitude linking Hyangnobong Peak, Mondong-ri, and Gimhwa-eup. Gangwon-do is 150 km wide, from east to west, and 243 km long, from north to south, and has an eastern coastline of approximately 314 km. The province is bordered by five provinces, three cities, and 13 counties. There are more than 140 mountains over 1000 m above the sea level (Figure 1).

### 2.3. HEMS and GEMS

The HEMS dispatch request is made through the long-distance hospital or a fire department call (119) requesting an emergency room transfer. All missions are communicated by the emergency physician, and after the patient's medical condition has been evaluated, a decision is made regarding a course of action. The mission request must also meet the approval of the helicopter pilot and aviation operator, who consider the aviation risks. The staff on board the helicopter include an emergency physician, a paramedic or nurse, a pilot, and a copilot. There is only one doctor-staffed helicopter in Gangwon Province.

GEMS include 119 fire department ambulances and private ambulances with a paramedic and a driver such as an EMT-Basic one driver on board. Ambulances are located in cities; in Gangwon Province, there are 49 fire department ambulances and 44 private ambulances.

During the study period, the means of transport of stroke patients to GEMS are classified into two categories: first, patients transferred by 119 ambulance, and second, patients transferred by private company ambulance.

### 2.4. Patients

**Figure 2:**
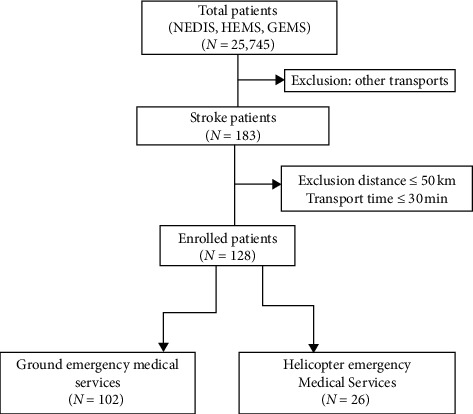
Flowchart of study patients. NEDIS: National Emergency Database Information System.

The stroke patients included in this study were over 15 years of age, with symptoms such as headache, disturbance of consciousness, convulsions, seizures, syncope, dizziness, numbness, and cerebrovascular relationship. We temporarily extracted all suspected cases and included in the final analysis patients classified as having a stroke according to the disease classification code ICD-I-639 (cerebral infarction). Patients included in the study were those with all available data, and patients were excluded if data were not available or if patient tracking was not possible (Figure 2).

### 2.5. Outcome Measures

Costs were analyzed by extracting all expenses of patients who were transported to the hospital and received medical treatment. The cost of all departments in which patients were treated and all hospital expenses (hospitalization expenses, surgery fees, and treatment expenses) were calculated. Six patients who were transferred to other hospitals were excluded. Emergency transportation costs included personnel expenses, activation allowances, insurance premiums, fuel costs, and equipment management expenses for each passenger patient transported.

### 2.6. Data Collection

Data for patients transferred to a regional emergency center via HEMS and GEMS were collected. HEMS data were obtained from the Gangwon HEMS team's patient transportation data and hospital materials (order communication system and electronic medical record), and the GEMS data were obtained from hospital records and the National Emergency Database Information system. For all patients and NEDIS (National Emergency Database Information System) data. In the study, cost calculations for patients were done by collecting hospital medical expenses (treatment, hospitalization, ICU, examination, national health benefit cost, personal medical insurance cost, and drug cost) and prehospital treatment and transportation costs.

### 2.7. Statistical Analysis

Patients were categorized into groups based on the mode of transport (HEMS versus GEMS) to the regional emergency medical center. Variables for each group are presented as frequencies and percentages for categorical variables and medians with interquartile ranges for continuous variables. Mean transportation times between groups were compared using Student's *t-*tests. A custom-fitted regression model was defined to compensate for the selection bias. We chose candidates by patient-level and hospital-level variables based on both face validity and associations observed in previous research [11, 12]. Patient-level factors included age, sex, vital signs, hospitalization period, transportation time, discharge rate, death rate, costs, and survival rate. Marginal associations between these variables and the outcome are summarized using proportions for categorical variables and means and medians for continuous measures. Statistical comparisons of observed values between outcome groups were based on weighted *χ*^2^ and *t*-tests adjusted for survey characteristics. If a continuous parameter was normally distributed, we applied the *t*-test for independent samples. For nonnormally distributed data, we used the Mann–Whitney *U* test. Pearson's *χ*^2^ test or Fisher's exact test was used to compare categorical variables. Standardized plausibility checks were carried out under statistical supervision. For statistical analyses of the data, we used IBM SPSS version 20.

### 2.8. Ethical Statement

This study was approved by the institutional review board of Wonju Severance Christian Hospital, Yonsei University (YWMR-15-5-043). Informed consent was waived by the board.

## 3. Results

In total, 7,580 patients were included in this study: of which 591 patients, including 178 patients with cerebrovascular disease, were transferred via HEMS and 6,989 patients, including 2,612 stroke patients, were transferred via GEMS. Patients with a diagnosis of ICD-I639 (cerebral infarction) were analyzed with respect to transfer time, survival rate, and cost-effectiveness. The mean time from the onset of the patient's symptom to hospitalization was 18.8 ± 126.1 minutes for HEMS and 335.2 ± 528 minutes for GEMS (*P*=0.001). The mortality rate was 1.73% for HEMS and 7.39% for GEMS, which was 4.23 times higher than for HEMS (*P*=0.001). We evaluated patients with the same disease classification code and, to reduce the study bias, patients were restricted to those aged between 60 and 80 years. The mean age was 69 years in the HEMS group and 67 years in the GEMS group. During the hospitalization period, only patients with a similar length of hospital stay (ICU or general ward) were included. Patients who were hospitalized for a period of time that was too short or too long were excluded. The total duration of hospitalization was 18.96 days in the HEMS group and 22.50 days in the GEMS group (*P*=0.848). The duration of ICU treatment was shorter in the HEMS group (5.22 days) than in the GEMS group (7.55 days). It can be inferred that there will be potential factors for active evaluation and management in HEMS transfer.

The transfer time from the field to the hospital was shorter for the HEMS group (1.12 hours) than for the GEMS group (1.56 hours) (*P*=0.001). The time from actual patient onset to hospital visit was 2.94 hours in the HEMS group and 4.20 hours in the GEMS group (*P*=0.001) (Table 1).

The time between arrival in the emergency room and the decision to admit to the hospital was 4.22 minutes in the HEMS group and 34.13 minutes in the GEMS group, indicating a significant difference in the time taken for evaluation and examination (*P*=0.001). The decision-making process was quicker for the HEMS group because the emergency physician actively evaluates and communicates on-site and during in-transit travel to request an appointment immediately upon arrival at the emergency room.

The difference in the discharge rate between the groups (96.2% in the HEMS group and 83.2% in the GEMS groups) was statistically significant (*P*=0.001). The mortality rate after 24 hours also showed a statistically significant difference of more than 4-fold between the groups: 3.8% in the HEMS group and 16.8% in the GEMS group (*P*=0.001) (Table 2). These results suggest that proper patient management by trained experts working within a system is likely to affect the patient's prognosis. None of the patients included in the study had died within 24 hours. The diagnosis of stroke is not always made in the hospital where initial treatment occurs, but the final diagnosis may be made in another hospital or by an insurance corporation. For this reason, it is inferred that there is no difference in the incidence of disability after the patient is released (*P*=0.803). According to the testimony of actual medical staff, it is necessary to improve the data management system in the future.

The cost of transferring one patient was calculated as $3,504 for HEMS and $262 for GEMS. Since the cost of HEMS and the operation cost of GEMS are operated as tax and local tax for all countries, there is no clear presentation method to calculate the amount of management expenses, labor costs, fuel costs, exercise allowance, and equipment. Hence, we calculated only the cost of one exercise such as administrative expenses. The average of overall hospital costs was calculated as follows: prehospital care expenses were calculated assuming that it is a one-time expense and hospital care expenses were calculated based on all costs incurred in hospitals such as those for emergency room treatment, ICU, general ward, rehabilitation, medical care, and a personal payment. We summed all expenditures and calculated the average for each group: $10,007 for the HEMS group and GEMS $10,141 for the GEMS group, with no statistically significant difference in total cost. Although the initial HEMS operating costs were high, the overall costs including hospital treatment costs did not differ significantly between the groups (*P*=0.978). There was a statistically significant difference in the survival rate after treatment: 96.2% in the GEMS group and 83.2% in the HEMS group (*P*=0.001).

Overall health care costs were higher in the HEMS group than in the GEMS group, but the difference was not statistically significant. The higher cost of HEMS is due to the high initial mobilization costs, which are necessary to save the patient's life. This can be considered an ethical question as to whether or not to improve the health care system. If it is to protect patient safety and save lives, it is worth doing.

## 4. Discussion

The purpose of this study was to analyze the effect of the HEMS and GEMS system, which are resources used in domestic EMS, on stroke patients. We found that the transportation via HEMS is quicker than that via GEMS, and the transport time to the hospital from the site, the entire time it took for the patient to arrive at the hospital after the time of the incident, and the time to determine whether a patient will be admitted or not to the hospital were all shortened, which were similar to the result of Ringburg et al. [13]. Indeed, our results are consistent with those of Eriksson et al. [14], who stressed that they found treatment in hospitals is faster, i.e., within 3 hours, which significantly influenced the patient's prognosis. Using GEMS Gangwon province with mountainous terrain can be a disadvantage for saving time. Therefore, the use of HEMS is more effective than GEMS in countries with mountainous terrain as in the study of Govindarajan et al. [15]. Rapid admission decisions in the emergency room are important for timely intensive care, and in our study, the admission decision time of patients transferred via HEMS was faster than that of patients transferred via GEMS. This is because the emergency medical specialists at the site and the regional hospital evaluate and treat the patient being transferred, and through communication with the medical staff at the hospital, preparations can be made for hospitalization ahead of time. From the prehospital stage, management of stroke patients reduces the delay in hospital treatment and enables rapid treatment [16]. For this process, accurate patient assessment by medical staff, medical communication, and an EMS delivery system is important [17–20]. The mortality rate of stroke patients transferred via HEMS was lower than that of patients transferred via GEMS. In the study of Konstantopoulos et al. [21], HEMS was effective for stroke patients and helped the systematic system operation of EMS.

In the study of Lukovits et al. [22], HEMS was found to be helpful in the transfer of stroke patients between hospitals from a wide range of provinces to urban areas. Xian [23] reported that transfer of stroke patients to the stroke center reduced the mortality and severity of their patients. A similar study suggested that professional and disciplined preparation of EMS would help to reduce patient mortality [24–26]. The use of HEMS is a powerful factor for patient transfer and treatment, and it is necessary to establish a continuous system for EMS system and country terrain [27]. In the studies of Ebinger et al. [28] and Fassbender et al. [29], thrombolysis therapy in the prehospital stage reduced the patient's treatment period. In our study, the entire treatment period of patients in the HEMS group was shorter than that of patients in the GEMS group, although the difference was not statistically significant. The cost of the prehospital stage and the overall medical cost including the treatment cost at the hospital did not show a big difference between HEMS and GEMS. In the end, the cost of HEMS management at the beginning of the prehospital stage can be high, but when looking at the overall medical expenses, costs are similar for HEMS and GEMS. In the study of Ringburg et al. [30], the overall medical expenses of HEMS and GEMS for trauma patients were highly analyzed via HEMS. In our study of stroke patients, the costs of HEMS and GEMS were at similar levels.

Overall, HEMS seems to be effective. Although the initial operating cost of HEMS seems to be higher than that of GEMS, it is in fact similar, because it decreases the mortality rate and increases the usual discharge rate. The Korean EMS system (GEMS) has been in operation for less than 20 years, and there is no clear direction regarding the management and transfer system of the stroke patient. Therefore, based on this research, we can expect to seek effective delivery methods for stroke patients. Considering the situation clarified in this study, we will advance to (A) stabilization and transfer; (B) stabilization, transportation, and notification; and (C) nerve evaluation, photography, treatment, cause classification, and simple notification. In order to operate the system stably, it is necessary to provide clear instructions for each transfer system. And, through ongoing research, it should be possible to clearly determine the decision to transfer patients.

### 4.1. Limitation

There is no linkage between prehospital data and hospital data. Further, data concerning the disability rate of stroke patients were not included in the study because these data are not available from the domestic records and are managed according to various conditions depending on individuals, regions, and affiliations. Additional research is required to validate the present findings by considering factors associated with patient management.

## 5. Conclusion

Compared with stroke patients transported via GEMS, those transported via HEMS received faster treatment, had a lower mortality rate at 24 hours after hospitalization, and had a higher discharge rate. These results are highly likely to enable rapid and accurate treatment through accurate judgment of emergency physicians and collaboration with medical staff at the hospital.

In conclusion, although HEMS require more initial investment and operation costs than GEMS, the average cost of the entire treatment, including the hospital stay, was similar to that of GEMS. Furthermore, there are many areas with mountainous terrain, where HEMS can be more effective than GEMS for longer range stroke patient transfer. Therefore, HEMS can be more effective than GEMS in areas with mountainous terrain and stroke patients that need to be transported over a long distance.

## Figures and Tables

**Table 1:** Characteristics of patients assisted via HEMS or GEMS.

	HEMS	GEMS	*P* value
Patients (male)	26 (7)	102 (65)	—
Age (mean ± SD)	69.70 ± 17.87	67.67 ± 13.48	0.608
Systolic BP	145.15 ± 32.88	148.38 ± 30.42	0.878
Diastolic BP	80.34 ± 24.37	83.76 ± 17.85	0.516
Pulse rate	78.88 ± 18.52	89.94 ± 19.04	0.024
Respiratory rate	18.73 ± 1.70	20.05 ± 3.60	0.020
Body temperature	36.51 ± 0.66	36.48 ± 0.94	0.881
Period of hospitalization (days)	18.96 ± 4.28	22.50 ± 4.14	0.584
ICU stay (days)	5.22 ± 0.99	7.55 ± 1.37	0.247
GW stay (days)	13.74 ± 4.03	14.95 ± 4.10	0.848

*Time analysis*			
Transportation time^*∗*^ (hours)	1.12 ± 0.68	1.56 ± 0.75	<0.001
Time from incident to GW admission (hours)	2.94 ± 1.91	4.20 ± 2.40	<0.001
Decision time for admission (min)	4.22 ± 1.93	34.13 ± 9.72	<0.001

HEMS: helicopter emergency medical services; GEMS: ground emergency medical services; ICU: intensive care unit; GW: general ward. ^*∗*^ Transportation time is the time of travel between the scene and the hospital.

**Table 2:** Discharge and death rate.

	HEMS	GEMS	*P* value
Discharge, *n* (%)	25 (96.2)	104 (83.2)	<0.001
Hopeless discharge, *n* (%)	0 (0)	1 (0.8)	0.608
Deaths after 24 hours, *n* (%)	1 (3.8)	21 (16.8)	<0.007
Deaths within 24 hours, *n* (%)	0 (0)	0 (0)	—
Patients transports (operation cost per transport) (USD)	$3,504	$262	—
Cost of hospitalization (USD) ^*∗*^	$10,007 ± $12,965	$10,141 ± $11,842	0.978
Survival rate (%)	96.2	83.2	<0.001

HEMS: helicopter emergency medical services; GEMS: ground emergency medical services. Current exchange rate: $1 = 1,141 Korean won.

## Data Availability

The data that support the findings of this study are available from the NEDIS but restrictions apply to the availability, which were used under license for the current study. They are not publicly available but are available from the corresponding author upon reasonable request.
